# Isolation and Identification of Two Clinical Strains of the Novel Genotype *Enterovirus E5* in China

**DOI:** 10.1128/spectrum.02662-21

**Published:** 2022-06-02

**Authors:** Chengyuan Ji, Yao Zhang, Ruini Sun, Zihao Pan, Jiale Ma, Huochun Yao

**Affiliations:** a MOE Joint International Research Laboratory of Animal Health and Food Safety, College of Veterinary Medicine, Nanjing Agricultural University, Nanjing, China; b Key Lab of Animal Bacteriology, Ministry of Agriculture, Nanjing, China; Changchun Veterinary Research Institute

**Keywords:** novel enterovirus, *Enterovirus E5*, cattle herds, novel genotype enterovirus

## Abstract

Most enterovirus (EV) infections are subclinical but, occasionally, can cause severe and potentially fatal diseases in humans and animals. Currently, EVs are divided into 12 types (A to L) based on phylogenetic analysis and on their natural hosts. Bovine enterovirus (BEV) is an essential member of the enterovirus belonging to the types E and F that attacks cattle as its natural host and causes clinical disorders in the digestive, respiratory, and reproductive tracts. In 2020, several dairy farms in China experienced cow mortality with acute clinical signs, including fever, and diarrhea. In these cases, GX20-1 and JS20-1 virus strains were isolated and sequenced. Cellular adaptation of these two strains showed efficient replications on Madin-Darby bovine kidney (MDBK) cells and produced a significant cytopathogenic effect (CPE). However, on baby hamster kidney (BHK-21) and Vero cells, viral replication was inefficient and did not produce CPE. As noted in comparative genomics analysis, these two strains showed distant evolutionary relationships with the well-known E1 to E4 and F1 to F4 subtypes of BEV and high sequence identities with the candidate type *Enterovirus E5*, a novel genotype recently identified based on the genomic data of three strains, including the GX20-1 and JS20-1 strains. This study provides the first evidence of a novel genotype bovine enterovirus infection in Chinese cattle herds, a potential threat to the cattle industry in China.

**IMPORTANCE** Bovine enterovirus (BEV) is a cattle-infecting pathogen. This study is the first report of natural infection of a novel genotype of enterovirus in herds of cattle in China. The homology of the novel enterovirus is far different from the structural protein of other enteroviruses and has different cellular adaptations. This study provides a reference for the biological characteristics and prevalence of the novel enterovirus in Chinese cattle populations.

## INTRODUCTION

The genus *Enterovirus* in the family *Picornaviridae* consists of 12 species of enteroviruses (*Enterovirus A*, *B*, *C*, *D*, *E*, *F*, *G*, *H*, *I*, *J*, *K*, and *L*) and three species of rhinoviruses (*Rhinovirus A*, *B*, and *C*) (1). Currently, the species of bovine enterovirus are classified as *Enterovirus-E* and *Enterovirus-F*, with clinical signs characterized by digestive, respiratory, and reproductive disorders (2–7). Bovine enterovirus (BEV) is a small, non-enveloped single-stranded positive-sense RNA virus containing a genome of approximately 7.3 to 7.5 kb with a unique open reading frame (ORF) flanked by two untranslated regions (UTRs) at the 5′ and 3′ ends. The ORF encodes a large polyprotein that is cleaved into four structural proteins and seven non-structural proteins. The P1 region of the viral polyprotein contains four structural proteins, VP4, VP2, VP3, and VP1, while the P2 and P3 regions contain seven non-structural viral proteins 2A, 2B, 2C, and 3A, 3B, 3C, 3D, respectively (8–13).

BEV was initially discovered in bovine, and subsequent prevalence studies proposed that cattle are the primary host reservoir for BEV worldwide. Moreover, BEV was found to infect a broad range of other animals, such as sheep, goats, horses, Australian brushtail possums, African buffalo, and impala (14–16). Furthermore, the BEV has zoontic potential as indicated by the recent demonstration of productive BEV replication in cells from diverse species and the high seroprevalence among humans, horses, dogs, sheep, and goats (17).

Bovine enterovirus classification has undergone a series of modifications. For instance, early attempts to classify BEV into seven serotypes were later revised to two serotypes (18, 19). Using a serological approach to type BEV is challenging due to cross-reactivity between BEV type-specific sera. However, due to the accumulation of BEV sequence data, BEV classification based on viral genetic variability and molecular differences has become feasible. For instance, based on the generally accepted definitions of *Picornavirus* species and serotypes, molecular-based BEV classification was performed by comparing the 5′-UTR sequences, identifying the capsid protein regions, and classifying bovine enteroviruses into *Enterovirus E* and *Enterovirus F* (20, 21). According to the International Committee on Taxonomy of Viruses (ICTV) criteria, members of species in the genus *Enterovirus* should have a high degree of aa identity (aa >70% in the polyprotein and aa >60% in P1) and compatibility in terms of handling, replication, and encapsulation (1).

This study identifies two enterovirus strains belonging to the novel subtype E5 recently identified, evaluates its phylogenetic and pathogenic characteristics, and reports this enterovirus as becoming endemic in Chinese cattle herds.

## RESULTS

### PCR detection of the virus.

To investigate the causative agents in these two cases (JS20-1 and GX20-1), cattle samples were collected for viral DNA or RNA extraction and were reverse transcribed into cDNA. Unexpectedly, the parasitological and bacteriological tests did not detect the potential pathogens. Moreover, virological detection, including BVDV-1, BVDV-2, BHV, BCoV, and BRV also showed negative results. Only the BEVs showed a weak positive result in one repetition of samples. To remove any potential doubts, the 5’UTR sequences of BEV were amplified. The sequencing results confirmed that BEV was the pathogen of the JS20-1 and GX20-1 cases.

### Genomic identification of the virus.

The BLAST analysis indicated that the sequences are highly related to the Enterovirus MexkSU/5 (KU172420), which belongs to the BEV genetic branch, but showed great evolutionary distances with subtypes BEVs, EV-E, and EV-F.

The MexkSU/5 was found by virome analysis of the nasal swab samples from the feedlot cattle with acute respiratory disease. Isolation of the live virus was not achieved (22). These results explain why the BEVs detection showed weakly positive results. The Enterovirus MexkSU/5 (KU172420) genome was used as the reference to design primer pairs for the whole-genome amplification of the GX20-1 and JS20-1 strains. The sequenced fragments were then assembled using the SeqMan and analyzed using the Megalign (DNAStar). The complete genomes of the two strains were obtained and submitted to the GenBank database under accession numbers MW477470 and MW579538, respectively.

### Observation of BEV particles by electronic microscopy.

**FIG 1:**
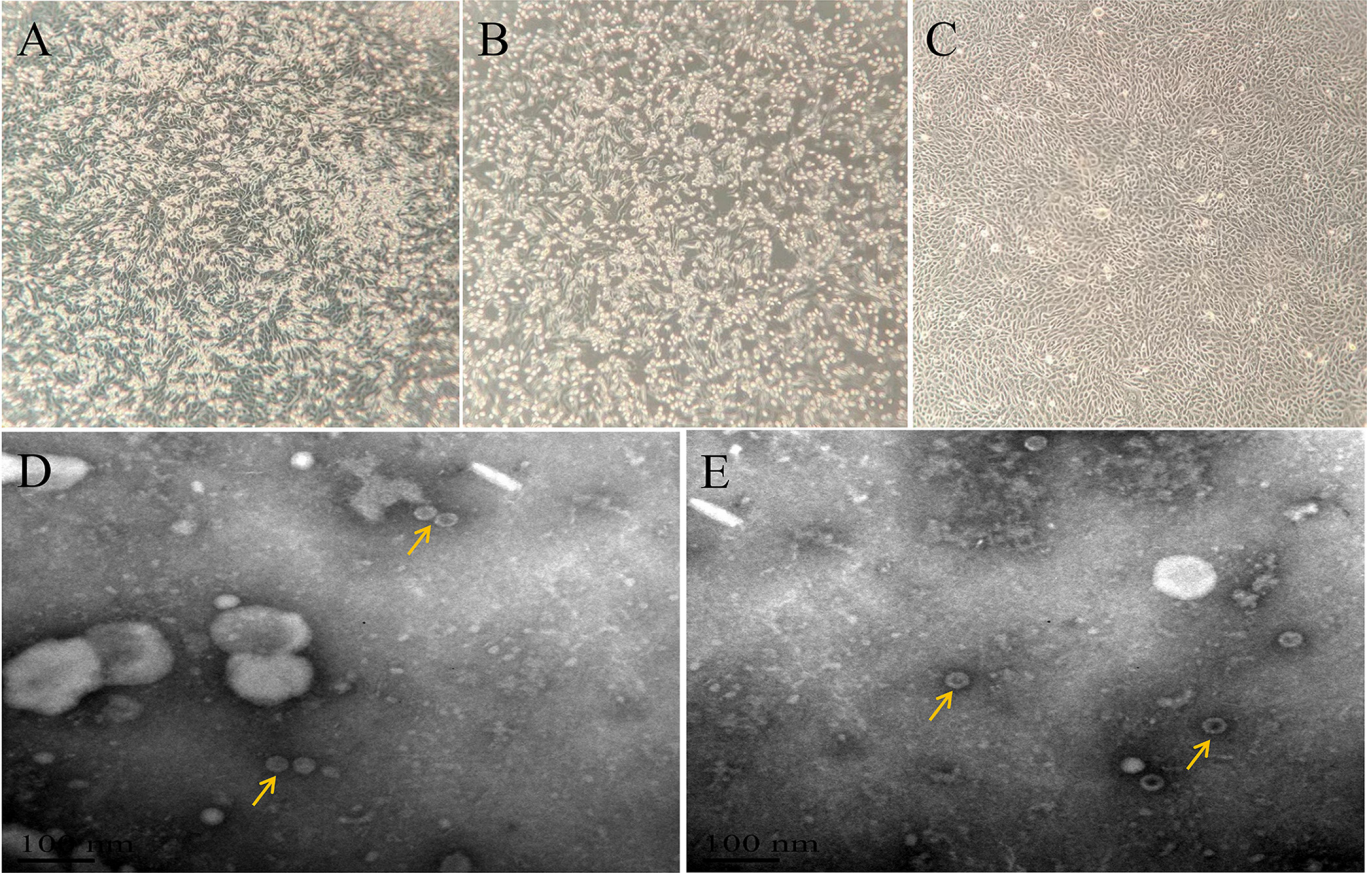
CPE and electron microscopic morphology of MDBK cells infected with BEV. (A) The CPE of MDBK inoculated with GX20-1. (B) The CPE of MDBK inoculated with JS20-1. (C) MDBK cells without virus inoculation (original magnification ×20). (D) The transmission electron micrograph of GX20-1. (E) The transmission electron micrograph of JS20-1.

Then, cell inoculation and replication of these viruses were carried out immediately. During the *in vitro* infection of Madin-Darby bovine kidney (MDBK) cells *in vitro*, the cytopathogenic effect (CPE) appeared after 36 h of inoculation for fecal samples (JS20-1, Fig. 1A) and the second generation of lung tissue samples (GX20-1, Fig. 1B) compared with the blank control cells (Fig. 1C).

Subsequently, a transmission electron microscope was employed to identify the samples. A large number of virus particles were observed in both strains, which showed uniform spherical particles with diameters of 20–30 nm (Fig. 1D, E).

### Infection characterization of the virus.

**FIG 2:**
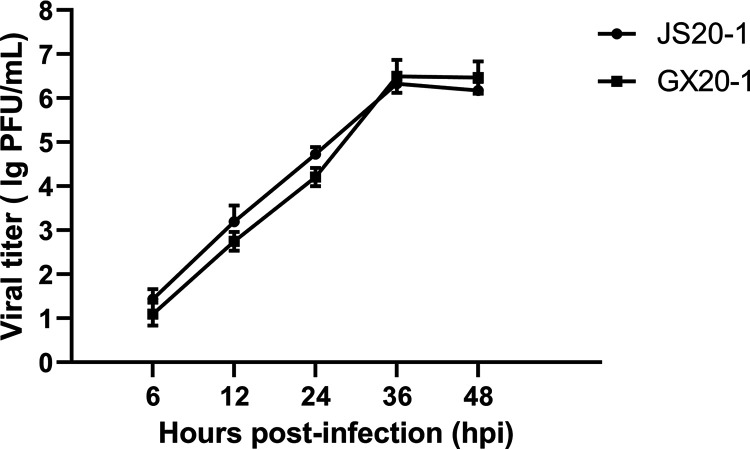
Growth kinetics of GX20-1 and JS20-1. MDBK cell monolayers were infected with GX20-1 and JS20-1 at an MOI of 0.05 and were harvested at 6, 12, 24, 36, and 48 hpi. Plaque assays were used to determine virus titers (PFU/mL) in MDBK cells in triplicate.

**FIG 3:**
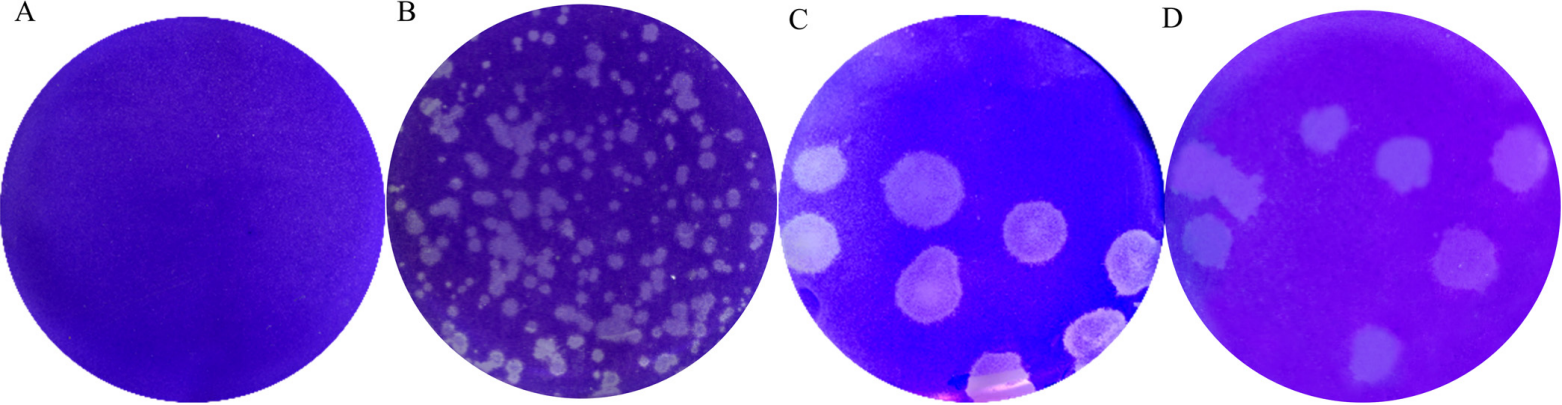
Plaque purification of BEV in MDBK cells. (A) Mock cells. (B) The BEV HB19-1 strain. (C) The GX20-1 strain. (D) The JS20-1 strain.

To detect virus growth kinetics, MDBK cells were inoculated with GX20-1 and JS20-1 at an multiplicity of infection (MOI) of 0.05 for the indicated time points. The virus titer presented a gradual upward tendency and peaked (GX20-1 was 4.4 × 10^6^ PFU/mL and JS20-1 was 3.3 × 10^6^ PFU/mL) at 36 h postinfection (hpi), showing that GX20-1 and JS20-1 strains could infect and replicate efficiently in MDBK cells (Fig. 2). Plaque assays showed that the plaques were approximately 3 to 4 mm in diameter after three purifications. Notable, these plaques are much larger in diameter than other species of bovine enterovirus (Fig. 3).

**FIG 4:**
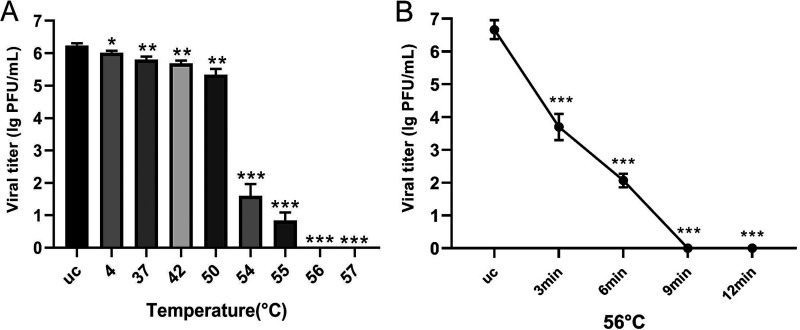
Temperature sensitivity of GX20-1. (A) Virus titers (PFU/mL) were determined in triplicate after treatment at 4 °C, 37 °C, 42 °C, 50 °C, 54 °C, 55 °C, 56 °C, and 57°C for 1 h. The unheated GX20-1 (uc) was used as the positive control. (B) Minimum time for inactivating GX20-1 at the most effective temperature. Virus titers (PFU/mL) were determined in triplicate after treatment at 56°C for 3, 6, 9, and 12 min. Experiments of temperature sensitivity were carried out independently at least three times, and the mean values of the log-transformed titers are shown ± the standard error of the mean (SEM). Differences in the titers were evaluated by a two-tailed *t* test with the thresholds of statistical significance as indicated (*, *P* < 0.05; **, *P* < 0.01; ***, *P* < 0.001).

**FIG 5:**
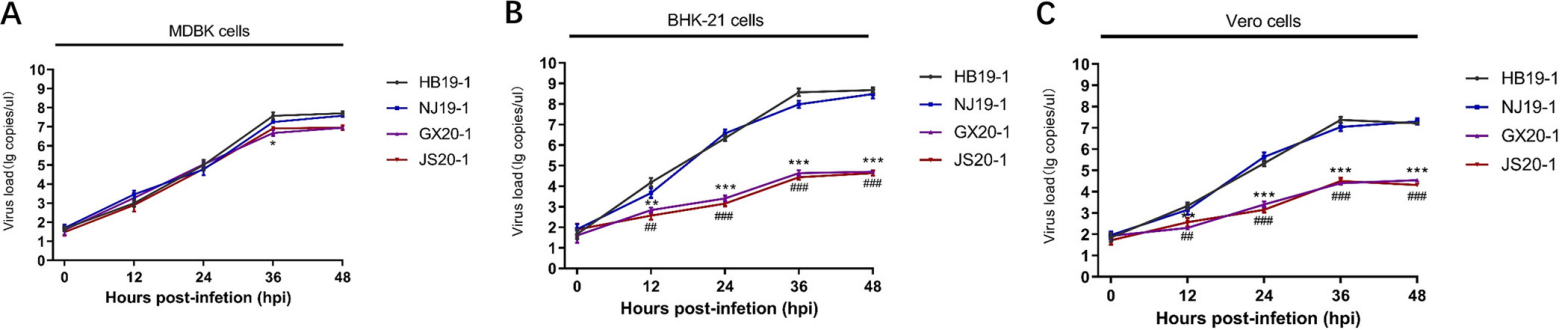
Viral RNA replication curves of the four BEV strains at 37°C. MDBK, BHK-21, and Vero cells were infected with HB19-1, NJ19-1, GX20-1, and JS20-1 at MOI = 0.05. RT-qPCR detected viral loads in the supernatants of infected cell cultures at indicated time points. At each time point, results are expressed as the mean ± SD of three independent experiments with triplicate samples. Using the two-way ANOVA test statistical analysis was performed with the Bonferroni correction. Legends: *, statistical analysis between GX20-1 and HB19-1 infected cells; #, statistical analysis between JS20-1 and HB19-1 infected cells. ** or #*#*, *P* < 0.01; *** or ##*#*, *P* < 0.001.

To detect temperature sensitivity, GX20-1 was heated at the indicated temperatures for 1 h and then used to inoculate MDBK cell monolayers. Compared with the untreated control, the GX20-1 infectivity decreased significantly after exposure to 37°C for 1 h. Moreover, with a continuous increase in treatment temperature, the virus titer continued to decrease (Fig. 4A). At the same time, GX20-1 was not inactivated completely at 55°C for 1 h (the viral titer was nearly 1 PFU/mL) but was inactivated completely at 56°C in only 9 min (Fig. 4B). Similarly, JS20-1 can be effectively inactivated under this condition. It is commonly known that bovine enterovirus with broad host tropism replicates efficiently on MDBK, baby hamster kidney (BHK-21), Vero, and many other cells. In contrast, GX20-1 and JS20-1 can only replicate efficiently on MDBK cells and multiply in BHK-21 and Vero, but with less efficiency and without producing CPE (Fig. 5).

### Phylogenetic analyses to identify viruses belonging to *Enterovirus E5*.

The following analysis indicates that both strains share the same genome organization with all enteroviruses, and contains a single large ORF comprising 6,549 bases that encode a predicted 2,182 aa polyprotein, VP1, P1, 3D, and polyproteins within this ORF. The BLAST analysis of entire genomes showed the highest sequence identity among strains GX20-1, JS20-1, and MexkSU/5 (KU172420).

A comprehensive analysis of the nucleotide and amino acid sequences of VP1, P1, 3D, and polyprotein for the GX20-1 strain were performed against other enteroviruses, including the JS20-1 isolate. Results indicated that the GX20-1 and JS20-1 isolates have the same evolutionary origin and showed the closest evolutionary distance with the MexkSU/5 strain (Table 1).

**TABLE 1:** Percent identity of nucleotide and amino acid sequences between GX20-1 and Enterovirus reference strains

		Identity (%)								
Strain	GenBank accession no.	VP1		P1		3D		Polyprotein		Subgenotype
		nt	aa	nt	aa	nt	aa	nt	aa	
JS20-1	MW579538	99.4	99.0	99.4	99.2	99.8	99.8	99.6	99.6	EV-E5
MexkSU/5	KU172420	91.0	97.3	90.5	97.0	92.5	98.3	91.3	98.1	EV-E5
VG27	D00214	53.0	51.5	61.6	64.1	82.9	96.3	72.5	82.2	EV-E1
PS42	DQ02792	53.3	52.0	62.6	65.7	79.7	95.0	71.8	82.3	EV-E2
HY12	KF748290	54.8	51.3	61.5	64.4	80.3	96.8	71.7	82.3	EV-E3
SL305	AF123433	53.5	53.8	62.2	64.7	81.0	97.2	72.0	82.9	EV-E4
261	DQ092770	54.1	57.5	62.8	66.9	71.8	83.1	65.2	82.9	EV-F1
PS89	DQ092795	55.0	56.4	62.5	66.2	71.4	83.1	64.9	73.2	EV-F2
PS87	AY508696	53.8	54.2	62.1	64.5	70.2	83.3	64.6	72.5	EV-F3
F4W1	AY462106	54.2	53.8	63.4	65.6	71.6	83.3	66.4	73.1	EV-F4
EV71	U22521	49.1	42.6	51.1	52.0	60.9	59.7	58.2	56.2	EV-A
EVB80	AY843298	46.9	40.4	49.0	47.7	66.1	70.9	58.3	57.6	EV-B
EVC13	DQ995644	44.5	37.3	49.2	45.2	64.7	67.0	56.9	53.9	EV-C
EVD	NC038308	48.1	39.3	53.0	50.9	62.2	63.9	57.7	55.2	EV-D
G3H	HQ705854	53.3	49.5	59.5	57.8	67.3	75.7	61.9	63.9	EV-G
SV4	AF326759	48.9	43.5	52.5	51.6	62.6	63.7	56.7	56.3	EV-H
N125	AF414372	50.3	44.7	56.1	54.7	64.2	67.9	58.8	59.5	EV-J

At the nucleotide level, the consistency of the Polyprotein segment, 3D gene, P1 segment, and VP1 gene of the GX20-1 strain was 64.6% to 72.5%, 70.2% to 82.9%, 61.5% to 63.4%, and 53.0% to 55.0%, respectively. At the amino acid level, the consistency was 73.1% to 82.9%, 83.1% to 97.2%, 64.1% to 66.9%, and 51.3% to 57.5%, respectively. These results indicate that the genomes of GX20-1, JS20-1, and MexkSU/5 showed great evolutionary distances from other enteroviruses (Table 1).

**FIG 6:**
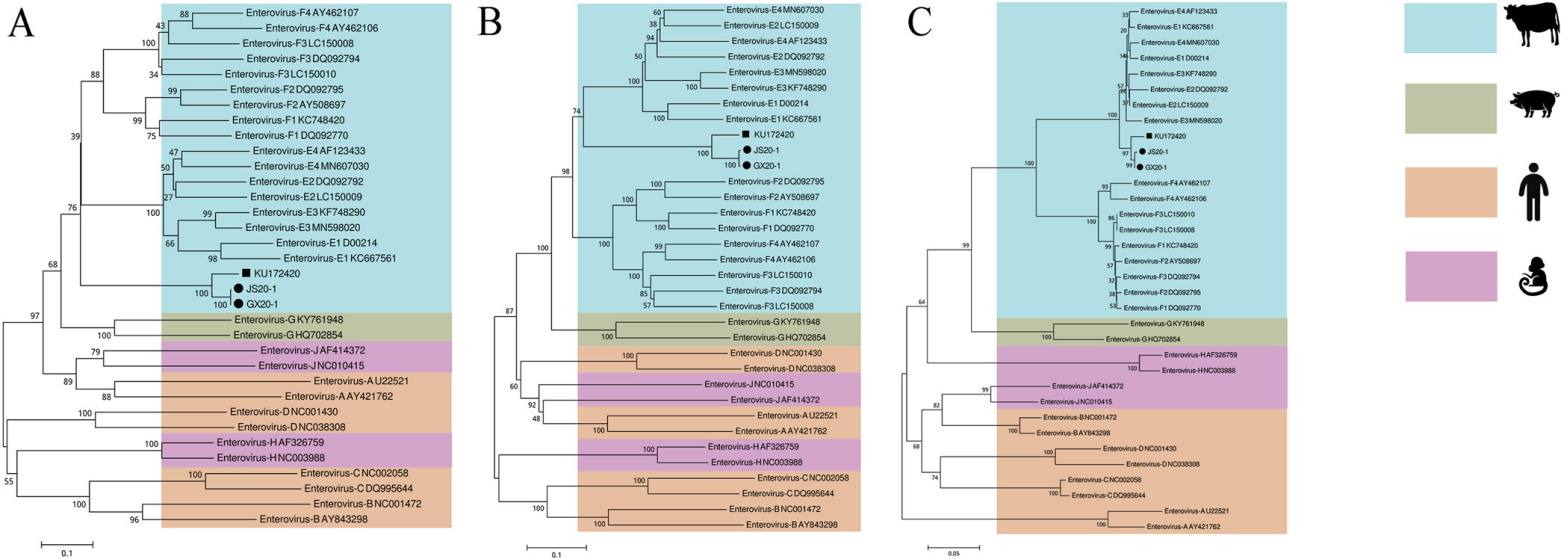
Phylogenetic trees based on the full-length amino acid sequence of different target genes from the GX20-1 and JS20-1, a novel bovine enterovirus (black dots); reference strain MexkSU/5 misclassified as candidate *Enterovirus E5* (black square); or retrieved from the National Center for Biotechnology Information Search database. A,VP1; B, P1; C, 3D.

Consistent with the preceding analyses, the GX20-1 and JS20-1 strains belong to the branch of bovine enterovirus. They are closely related to the MexkSU/5 strain in our four phylogenetic trees, constructed based on the VP1, P1, and 3D amino acid sequences from diverse enteroviruses, respectively (Fig. 6).

Significantly, the GX20-1, JS20-1, and MexkSU/5 strains are located at an independent branch in each tree. In the VP1 and P1 trees, these three strains failed to cluster with EV-E or EV-F, only the 3D protein tree was found to cluster closely to EV-E reference sequences.

Further analyses showed that the GX20-1, JS20-1, and MexkSU strains shared less than 67% sequence identity with other BEVs in proteins P1 and VP1; this is significantly lower than the dividing line at 70% for heterologous serotypes. Although Namita Mitra et al. (22) defined MexkSU/5, a virome-sequenced by genome, as an EV-E4 strain, our phylogenetic tree showed that these three strains formed an independent branch but failed to cluster with any others of the previous bovine enteroviruses. These results suggest that the GX20-1 and JS20-1 are taxonomically distant from previously reported BEVs based on the taxonomic definition. Taken together, we proposed these two strains to be classified as a new phylogenetic type of enterovirus, and submitted their genomes to the GenBank database. Subsequently, the *Picornaviridae* website designated the novel subtype *Enterovirus E5* based on the genomic data of the GX20-1, JS20-1 and MexkSU/5 strains (https://www.picornaviridae.com/ensavirinae/enterovirus/ev-e/ev-e_seq.htm).

### Recombination analysis of GX20-1 to explore the potential evolutionary process of *Enterovirus E5*.

**FIG 7:**
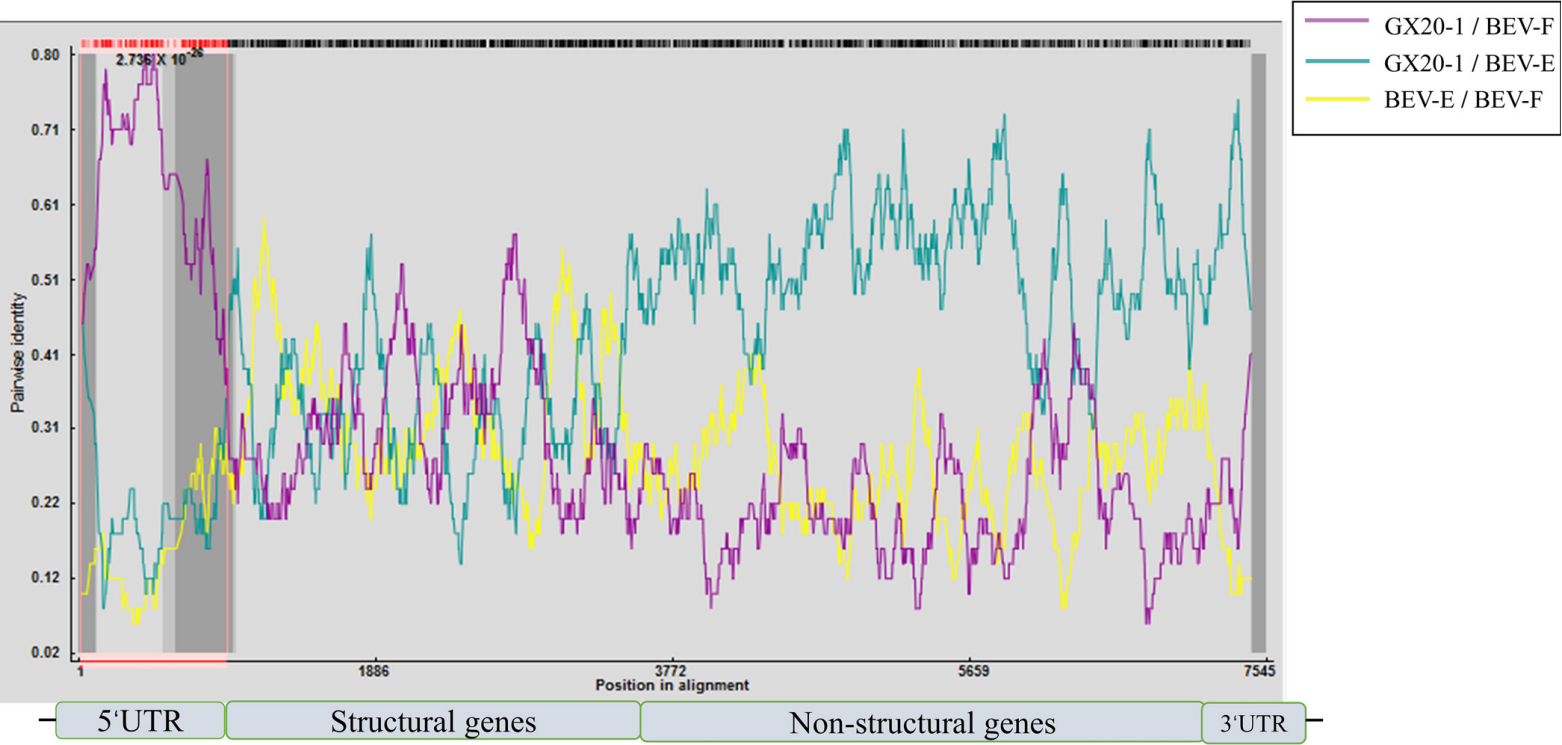
Recombinations within the GX20-1 genome. Recombination analysis performed by RDP4. Curves display whole-genome pairwise identities of BEV-E/BEV-F (yellow), GX20-1/BEV-E (cyan), and GX20-1/BEV-F (purple).

The complete genome sequences of BEV strains were inputted to RDP4 to search for recombination signals. Detailed sequence analysis was conducted to investigate the possible virus recombination events and indicates that the putative recombination site was located at nucleotide position “945,” which separates the GX20-1 genome into two parts. The sequence identity between BEV-E and GX20-1 in the region of the non-structured genes was significantly higher than that of BEV-F (Fig. 7).

### Epidemiological investigation of *Enterovirus E5* infection in the farms.

A real-time RT-PCR assay using a pair of primers and a probe specifically targeting the 5’UTR region of BEV species was performed to analyze the prevalence characteristics of BEV by detecting bovine feces samples from various provinces in China. The positive BEV rate in these feces was 27.6% (34/123). However, the positive rate of *Enterovirus E5* was only 1.6% (2/123). One E5 sample was collected from the Guangxi province and another from Shandong Province.

## DISCUSSION

Generally, BEV infection-dependent clinical symptoms are characterized by diarrhea, respiratory disorders, and abortion; rare fatal cases with low morbidity have been reported (23). However, as increasing instances of BEV isolates are identified from fatal intestinal and respiratory diseases, the pathogenicity and virulence of BEV have reemerged with some variations. Numerous in-depth studies explored its relevance to disease (2, 22, 24–28).

This study reports an acute and lethal case in cattle infected by a novel genotype of bovine enterovirus in China, which was recently designated as an E5 subtype. The molecular definition of *Picornavirus* genotypes is determined by the diversity of capsid proteins. Moreover, less diverse non-structural protein regions are used to define enteroviruses.

The GX20-1and JS20-1 strains share a 64% to 67% sequence identity with other BEVs in proteins P1, as well 72% to 83% in the polyprotein, as per ICTV classification criteria (aa >70% in the polyprotein and aa >60% in P1), thus forming an independent branch in all phylogenetic analyses, including VP1, P1, and 3D, suggesting that GX20-1 and JS20-1 comprise a novel genotype within the genus *Bovine enterovirus*. Notably, this strain was isolated from two different provinces, thus indicating that this genus of strain is gradually evolving and spreading in dairy farms and possibly becoming endemic in Chinese cattle farms. This scenario poses a new challenge for preventing and controlling cattle diseases and potentially emerging viruses.

Recently, the *Picornaviridae* website designated the novel subtype *Enterovirus E5* based on the genomic data of GX20-1, JS20-1, and MexkSU/5, indicating that the two strains identified by this study have been conferred a degree of official acceptability as original isolates of this novel genotype of Enterovirus.

In the results of this study, the *Enterovirus E5* strains were only found in cattle and showed a specific viral tropism to cattle. The potential reasons associated with this characteristic are that the *Enterovirus E5* strains have circulated among cattle for a long time, and some viruses have evolved to adapt to cattle and therefore have a greater chance of replicating in them. China is a densely cattle-populated country. A large number of cattle can quicken the viral spread. Thus, the altered host tropism (cattle-adapted) can promote the spread of BEVs.

Do BEV strains from the novel genotype show infection characteristics that are different from traditional BEV isolates? An apparent tissue predisposition characterizes most viruses due to the presence of specific cell surface receptors. However, BEV is an exception. BEV is routinely cultured in BHK-21 cells, yet is commonly known to be readily adaptable to grow in the HeLa, human cervical carcinoma cell line, to equivalent titer (29). Moreover, the BEV can also be adapted to grow on different cell lines from porcine, human, or monkey origin. So, in the case of BEV, the receptor may be a ubiquitous cell surface glycoprotein. Although attempts have been made to identify BEV cell surface receptors, these efforts are hampered by the wide range of cell types in which cytopathic effects may be demonstrated *in vitro*. Unexpectedly, the two isolates of GX20-1 and JS20-1 have limited host adaptions. Thus, we posit that their surface features are not similar to those of other BEVs. In addition, growth curves for both strains in bovine mammary epithelial cells (MAC-T cells) showed results similar to those on MDBK (data not provided). Both strains appear to have altered tropism and could replicate well in cells of bovine origin, similar to BEV other genotypes. However, they were poorly adapted in cells other than those of bovine origin.

In summary, this study describes the first clinical cattle case of a natural, novel phylogenetic type of bovine enterovirus infection, recently designated as *Enterovirus E5*. In most countries the epidemiological status and virus variation of the bovine enterovirus remain unclear. Thus, this emerging phylogenetic type of bovine enterovirus could present a significant threat to the cattle industry in China and worldwide.

## MATERIALS AND METHODS

### Sample collection.

In January 2020, a dairy farm in the Guangxi Province of China within our pathogen monitoring system reported a GX20-1 case of a calf that died after a 5-day medical treatment. The calf showed acute clinical symptoms such as high fever, diarrhea, anorexia, and depression. The main pathological change was extensive edemas in the two lungs. In April 2020, an adult cow from a dairy farm in the Jiangsu Province of China was reported dead with similar symptoms (JS20-1). Laboratory diagnosis was performed on these two cases (GX20-1 and JS20-1).

### Nucleic acid extraction and PCR.

Viral RNA was extracted from the sample using the QIAamp Viral RNA minikit (Qiagen, Germany). The first strand of cDNA was synthesized by reverse transcription using the HiScript II 1st Strand cDNA Synthesis Kit (Vazyme, China) with oligo (dT) primers. Bovine viral diarrhea virus 1 and 2 (BVDV-1 and BVDV-2), BEV, bovine rotavirus (BRV), bovine coronavirus (BCoV), and bovine rhinotracheitis virus (BHV) were examined by RT-PCR or regular PCR with specific primers as described (30–32). PCR products were subjected to electrophoresis with 1.5% agarose gel, visualized with UV light (Bio-Rad, USA), and/or purified for DNA sequencing (Genewiz, Suzhou, China). Sequences were compared with existing sequences in databases using the Basic Local Alignment Search Tool (BLAST).

### Cell culture and viruses.

MDBK, BHK-21, and Vero cells were cultured in Dulbecco’s Modified Eagle’s Medium (Gibco) and supplemented with 10% fetal bovine serum (Gibco) and 1% penicillin-streptomycin at 37°C with 5% CO_2_. The BEV-E NJ19-1 strain and BEV-F HB19-1 (GenBank accession no. MW468092) are from the laboratory reserve.

### Virus isolation.

Centrifugation at 12,000 × *g* for 10 min at 4°C was used to prepare 10% lung tissue homogenate and fecal samples. The supernatants were filtered with 0.22 μm filters, and the flow-through was used to inoculate MDBK cells. After three consecutive passages, viruses were harvested by three freeze-thaw cycles and were further confirmed by RT-PCR.

### Growth characterization and temperature sensitivity *in vitro*.

Viral plaque assays were performed using MDBK cells grown in six-well plates. Viral samples were serially 10-fold diluted in DMEM. At least 500 μL of each diluted sample was inoculated onto monolayers of MDBK cells and incubated for 1 h. The cells were then overlaid with a mixture of DMEM containing 1% low-melting agarose (Cambrex, Rockland, ME, USA) and incubated at 37°C for 3 days in 5% CO_2_. After removing the medium, the cells were stained with 1 to 2 mL of a staining solution consisting of 0.5% crystal violet and 25% formaldehyde solution. Then, the plaques were counted, and the virus titers were expressed as PFU/mL.

In addition, infected cells were collected at 0, 12, 24, 36, and 48 hpi. Viral loads were determined by a universal real-time qRT-PCR assay. To further examine temperature sensitivity, GX20-1 was heated at 4 °C, 37 °C, 42 °C, 50 °C, 54 °C, 55 °C, 56 °C, and 57°C for the indicated time. Then, virus titers (PFU/mL) were determined in triplicate in MDBK cells.

### Preparation of virus particles for electron microscopy observation.

The MDBK cell monolayers were infected with GX20-1 at an MOI of 1 for 24 h. The cells were subjected to three freeze-thaw cycles. The cellular debris was clarified by centrifugation at 10,000 × rpm at 4°C for 30 min. The crude virus was pelleted from the clarified supernatant by ultracentrifugation at 2,5000 × rpm at 4°C for 2 h. Then, the virus pellet was resuspended in 0.5 mL PBS and then layered onto a 20% to 60% (wt/vol) discontinuous sucrose solution by centrifugation at 2,8000 × rpm at 4°C for 2 h. Transmission electron microscopy (TEM) was used to detect and collect the virus band at the interface.

### Phylogenetic analysis and sequence alignments.

Full-length genomes from the isolated bovine enterovirus strains were amplified by RT-PCR and sequenced for phylogenetic analysis. Primer sequences are listed in Table 2. The neighbor-joining phylogenetic trees of VP1, P1, and 3D were constructed based on a ClustalW alignment using the MEGA 7.0 software with 1,000 bootstrap replicates. All reference sequences were collected from the GenBank database.

**TABLE 2:** Primers for complete genome sequence amplification

Name	Forward primer (5′–3′)	Reverse primer (5′–3′)	Position
MexKSU-1F/R	TTTAAAACAGCTCGGGGGTTGTTCCC	TCACTGTATCCACATGCTTCAGCA	1 to 1060
MexKSU-2F/R	CAGCCTATTGCAGATGTGATCAA	CGACCTGTACAATTTCTAAGAG	1003 to 1908
MexKSU-3F/R	CCTGAGATTTTTATACCAGGAGAAGTG	CCTGGTGGTACATACATGACTTG	1880 to 2962
MexKSU-4F/R	CATGCGTTTTGACCTTGAATTCAC	AGCTGCGTATAATGGTGCGCTCTTCC	2872 to 4369
MexKSU-5F/R	GAACATTCCTCTGCAAGCCAAGA	CTGGTATCATTGCTCTGATATCTC	4318 to 5706
MexKSU-6F/R	GAATGATAAAACTGATACCTCCC	ACACCCCATCCGGTGGGTGTATTTA	5640 to 7435

### Recombination analysis.

Genomic sequences of GX20-1 and other BEV strains were aligned using RDP4 to detect possible recombination events. Seven methods were applied, including RDP, GENECONV, Chimaera, MaxChi, 3Seq, SiScan, and BootScan (33). At least six methods with *P* values less than 0.05 were required for sequences to be considered recombinant and subject to further analysis. The parameters were set with a window size of 500 bp and a step size of 50 bp.

### Retrospective research.

To investigate the prevalence of *Enterovirus E5* on the cattle farms, 22 cattle droppings were obtained again from the Jiangsu farm and 20 from the Guangxi farm. In addition, fecal samples from Shandong (*n* = 24), Inner Mongolia (*n* = 20), and Hebei (*n* = 37) were obtained for the identification of *Enterovirus E5*. Based on the Taq-Man Real-time qPCR method, these 123 samples were tested for BEV, and the positive samples were further sequenced (21).

### Statistical analysis.

Summary statistics were calculated to assess the overall quality of the data. All data were processed using the GraphPad Prism software (version 8.0). The experiments *in vitro* were independently carried out at least three times. The *t* test was used to evaluate the statistical significance of the virus titers. Statistical significance was set to a *P*-value < 0.05.

### Data availability.

The complete genomes of the two strains were obtained and submitted to the GenBank database under accession numbers MW477470 and MW579538. All other data generated or analyzed during this study are included in this article.
